# Voxel-based and surface-based morphometric analyses reveal cortical–subcortical structural abnormalities and cognitive correlates in drug-naïve SeLECTS

**DOI:** 10.3389/fneur.2026.1769284

**Published:** 2026-04-16

**Authors:** Jiaren Zhang, Maoqiang Tian, Linfeng Song, Xu Chen, Xuejin Ma, Tijiang Zhang, Lin Jiang

**Affiliations:** 1 Department of Radiology, The First People’s Hospital of Zunyi, The Third Affiliated Hospital of Zunyi Medical University, Zunyi, Guizhou, China; 2 Department of Pediatric Neurology, The Affiliated Hospital of Zunyi Medical University, Zunyi, Guizhou, China; 3 Department of Radiology, The Affiliated Hospital of Zunyi Medical University, Zunyi, Guizhou, China

**Keywords:** asymmetry, cognitive dysfunction, pons, self-limited epilepsy with centrotemporal spikes, surface-based morphometry, voxel-based morphometry

## Abstract

**Background:**

Self-limited epilepsy with centrotemporal spikes (SeLECTS) is a common childhood epilepsy syndrome characterized by spontaneous seizure remission but frequent cognitive difficulties. Previous neuroimaging studies have reported cortical abnormalities in SeLECTS; however, findings remain heterogeneous and are often confounded by antiseizure medications exposure or by reliance on a single morphometric approach.

**Methods:**

We conducted a multimodal structural MRI study on 30 drug-naïve children with SeLECTS and 30 age- and sex-matched healthy controls. Voxel-based morphometry was used to quantify gray matter volume, surface-based morphometry was employed to assess cortical thickness, gyrification, and sulcal depth, and a lateralization index was used to evaluate hemispheric asymmetry. Exploratory correlation analyses were performed between these results and clinical variables as well as scores from the Wechsler Intelligence Scale for Children-Revised.

**Results:**

Patients showed increased bilateral pontine gray matter volume compared to controls. SBM identified widespread cortical thinning in frontoparietal and left temporal regions, increased gyrification in the right lateral orbitofrontal and left superior frontal gyri, and reduced right medial temporal sulcal depth. Atypical leftward lateralization was observed in the supramarginal, angular, and middle occipital gyri. Right pontine volume positively correlated with disease duration, while left superior frontal gyrification negatively correlated with verbal IQ.

**Conclusion:**

Drug-naïve children with SeLECTS exhibit a complex pattern of cortical dysmaturation and subcortical structural variations. These findings suggest that the neuroanatomical signature of SeLECTS extends beyond the Rolandic cortex, involving subcortical nuclei and widespread developmental pruning pathways. While the mechanistic links to cognition remain speculative, these structural markers provide a framework for future longitudinal studies.

## 1 Introduction

Self-limited epilepsy with centrotemporal spikes (SeLECTS), formerly termed benign epilepsy with centrotemporal spikes, is the most common focal epilepsy syndrome of childhood, accounting for approximately 10–20% of pediatric epilepsies (1, 2). The disorder typically presents between 3 and 14 years of age and is characterized by centrotemporal epileptiform discharges and an age-dependent remission of seizures during adolescence (3). Despite this favorable seizure prognosis, accumulating evidence indicates that many children with SeLECTS experience persistent difficulties in language, attention, and executive function (2, 4, 5). This dissociation between seizure and neurodevelopmental outcomes prompted the International League Against Epilepsy (ILAE) to revise its terminology from “benign” to “self-limited” (1), reflecting an updated understanding based on evidence of neurocognitive risk. The underlying neurobiological mechanisms remain debated, with two primary models: the “spike-impact” model, where frequent discharges disrupt functional networks (6), and the “dysmaturation” hypothesis, suggesting an atypical trajectory of cortical development (7, 8).

Neuroimaging offers a noninvasive means of investigating these mechanisms. However, prior studies have yielded inconsistent findings, likely due to methodological heterogeneity, small sample sizes, and the confounding effects of antiseizure medications (ASMs) exposure, which can obscure the primary neurobiological features of the syndrome. Medications like valproate have been linked to significant reductions in gray matter volume and cortical thickness, potentially masking the primary effects of the epilepsy (9, 10). In contrast, levetiracetam has been suggested to have a potentially normalizing effect on Rolandic cortices (11).

Voxel-based morphometry (VBM) has been widely used to examine gray matter (GM) volume alterations in SeLECTS and related epilepsy syndromes, with reported abnormalities in frontal, temporal, cingulate, and Rolandic regions (12–14). While VBM provides a whole-brain, voxel-wise assessment of volumetric differences, it is limited in characterizing cortical geometry. Its principal strength, however, lies in detecting volumetric differences in subcortical structures that are inaccessible to surface-based techniques. Meanwhile, abnormal asymmetry in brain structure is considered to be associated with cognitive function specialization and neurodevelopmental disorders (15, 16). Abnormalities in brain structural asymmetry have been reported in conditions such as attention-deficit/hyperactivity disorder, autism spectrum disorder, and dyslexia, but have not been fully explored in SeLECTS (15, 17, 18).

Surface-based morphometry (SBM) complements VBM by enabling direct measurement of cortical thickness (19), sulcal depth (12), and gyrification (20). These surface-derived metrics are sensitive to neurodevelopmental processes such as synaptic pruning, dendritic arborization, and cortical expansion, and may therefore capture aspects of cortical dysmaturation that are not reflected in volumetric analyses alone. In typical development, cortical thickness increases until late childhood and then declines during adolescence as excess synapses are pruned (21). In contrast, cortical gyrification is largely established prenatally and in early childhood, with only minor reductions during later childhood and adolescence (22). Sulcal depth, which is closely related to cortical folding, undergoes its most rapid changes in the perinatal period and remains relatively stable thereafter. Multimodal approaches integrating VBM and SBM have shown particular promise in pediatric neuroimaging by allowing simultaneous assessment of cortical and subcortical structures (23).

In the present study, we apply an integrated VBM and SBM approach to a cohort of 30 drug-naïve children with SeLECTS and 30 healthy controls. Our primary hypothesis-driven aims are to: (1) determine if cortical thinning occurs in the frontoparietal regions, potentially reflecting aberrant pruning, and (2) characterize hemispheric asymmetry in perisylvian language hubs. We also conducted exploratory whole-brain analysis to identify subcortical variations, specifically within the brainstem nuclei, and evaluate their relationship with disease duration and cognitive performance. By studying a medication-free cohort, we aim to provide a clearer characterization of the structural brain correlates that define this common childhood epilepsy syndrome.

## 2 Materials and methods

### 2.1 Study design and participants

This cross-sectional study consecutively recruited 60 right-handed children between August 2019 and August 2024, including 30 drug-naïve patients with SeLECTS and 30 age- and sex-matched healthy controls (HCs). All participants were recruited at the Affiliated Hospital of Zunyi Medical University, Zunyi, China. The diagnosis of SeLECTS was established according to the ILAE classification criteria (24). All patients underwent interictal electroencephalography (EEG) recording, which included natural sleep or drowsiness whenever possible to maximize the detection of centrotemporal spikes.

Inclusion criteria for the SeLECTS group were: (i) focal motor or focal-to-bilateral tonic–clonic seizures predominantly occurring during sleep; (ii) electroencephalographic evidence of centrotemporal spikes; (iii) age between 6 and 14 years; (iv) full-scale intelligence quotient (FSIQ) ≥ 70; (v) no prior exposure to ASMs. Exclusion criteria for all participants included: (i) visible structural brain lesions on conventional MRI (T1-weighted or FLAIR); (ii) history of neurological or psychiatric disorders other than SeLECTS; (iii) excessive head motion (>3 mm translation or >3° rotation); (iv) inadequate image quality.

### 2.2 Cognitive and clinical assessment

The cognitive assessment of the SeLECTS group was conducted using the Chinese version of the Wechsler Intelligence Scale for Children-Revised (WISC-RC). This tool was selected based on its established regional standardization and validity within the Chinese clinical setting. The Full-scale IQ (FSIQ), Verbal IQ (VIQ), and Performance IQ (PIQ) were recorded to allow for exploratory correlation analyses with the structural findings. All MRI scans and neuropsychological tests were completed within a single day, with MRI scanning consistently performed prior to cognitive testing to avoid potential fatigue effects on neuropsychological performance. Clinical data, including age of onset, seizure frequency, and duration of epilepsy, were collected through retrospective medical record review. Formal cognitive testing was not performed in the control group, which is acknowledged as a limitation when interpreting structure–cognition relationships.

### 2.3 MRI data acquisition

All participants underwent MRI scanning on a 3.0 Tesla GE HDxt scanner (GE Healthcare, USA) equipped with an 8-channel head coil. High-resolution three-dimensional T1-weighted images were acquired using a Brain Volume Imaging (BRAVO) sequence with the following parameters: repetition time = 1900 ms, echo time = 2.1 ms, inversion time = 900 ms, flip angle = 9°, matrix size = 256 × 256, voxel size = 1 × 1 × 1 mm^3^, and slice thickness = 1 mm. Foam padding was used to minimize head motion during scanning.

### 2.4 Voxel-based morphometry

VBM was performed using the Computational Anatomy Toolbox (CAT12, version 12.8) implemented in SPM12 (Wellcome Trust Centre for Neuroimaging, London, UK) running in MATLAB 2024b. T1-weighted images were segmented into gray matter (GM), white matter, and cerebrospinal fluid using tissue probability maps. Images were spatially normalized to Montreal Neurological Institute (MNI) space using DARTEL, modulated to preserve volume information, and smoothed with an 8-mm full-width at half-maximum (FWHM) Gaussian kernel. To ensure the reliability of the statistical analysis, absolute threshold masking was set to 0.2. According to the CAT12 manual (25), increasing the threshold to 0.2 is utilized to effectively exclude non-brain areas that might otherwise inflate false-positive rates, while still preserving deeper subcortical and brainstem gray matter voxels. Segmentation accuracy was verified using the CAT12 quality control report, which calculates a weighted image quality rating based on noise and inhomogeneity; only images achieving a quality rating higher than B were included in the final analysis. Total intracranial volume (TIV), age, and sex were included as covariates. Statistical significance for VBM was assessed using family-wise error correction at the voxel-wise (*p* < 0.05) and a cluster extent threshold of 20 voxels.

To provide interpretive context for regions showing significant VBM differences, exploratory meta-analytic decoding was performed using the Neurosynth platform (see Supplementary methods in Appendix). This procedure involves a spatial correlation between the study’s group-level statistical map and term-based meta-analytic activation maps derived from thousands of published MRI studies. These results are presented purely for hypothesis generation and do not support anatomical or functional localization in the current sample.

### 2.5 Structural lateralization analysis

Hemispheric structural asymmetry was quantified using a lateralization index (LI), calculated for homologous regions defined by the Desikan–Killiany atlas. The LI was defined as:

$$\mathrm{LI}=\frac{V_{L}-V_{R}}{V_{L}+V_{R}}$$

Where 𝑉_𝐿_ and 𝑉_𝑅_ represent regional morphometric values in the left and right hemispheres, respectively. In this analysis, 𝑉 represented the gray matter volume of each region (in cubic millimeters). Positive values indicate leftward dominance (𝑉_𝐿_ > 𝑉_𝑅_), while negative values indicate rightward dominance (𝑉_𝑅_ > 𝑉_𝐿_).

To reduce noise from regions with minimal asymmetry, regions showing < 5% baseline asymmetry in healthy controls were excluded from group-level LI comparisons. Between-group differences in LI were assessed using independent-samples *t*-tests with false discovery rate (FDR) correction for multiple comparisons. All lateralization analyses inherently focused on cortical gray matter, as V represents GM volume per region; white matter was not included.

### 2.6 Surface-based morphometry

SBM analyses were conducted using CAT12 following standard preprocessing pipelines. Cortical surfaces were reconstructed from T1-weighted images, and cortical thickness, gyrification index, and sulcal depth were extracted for each participant based on the Desikan-Killiany atlas (26). Cortical thickness was calculated as the distance between the gray-white matter boundary and the pial surface, smoothed with a 15-mm FWHM kernel. Gyrification index was computed using a curvature-based approach reflecting local cortical folding complexity, smoothed with a 20-mm FWHM kernel (27). Sulcal depth was estimated as the distance from the cortical surface to the sulcal fundus and smoothed with a 20-mm FWHM kernel (28). Group comparisons were performed using general linear models with age and sex as covariates. Multiple comparisons were controlled using FWE correction at *p* < 0.05.

### 2.7 Statistical analysis

Demographic and clinical variables were analyzed using SPSS version 29.0 (IBM Corp., Armonk, NY, USA). Group differences in continuous variables were assessed using independent-samples *t*-tests, and categorical variables were compared using chi-square tests. For neuroimaging analyses, age, sex, and TIV were included as covariates where appropriate. Correlations between imaging metrics and clinical or cognitive variables were tested using Pearson’s correlation coefficient. Given the sample size and number of comparisons, these correlation analyses (including specific assessments of cognitive subscores such as VIQ) were not pre-specified endpoints but were considered exploratory analyses, and the results should be interpreted with caution. Statistical significance was set at *p* < 0.05, with FWE or FDR correction applied for neuroimaging analyses unless otherwise stated.

## 3 Results

### 3.1 Comparison of sociodemographic and clinical characteristics

Demographic and clinical characteristics of the SeLECTS and HC groups are summarized in Table 1. There were no significant between-group differences in age (*p* = 0.81), sex distribution (*p* = 0.30), or years of education (*p* = 0.59). Handedness was assessed using the Edinburgh Handedness Inventory, confirming that all 60 participants were right-handed. All patients in the SeLECTS group were drug-naïve at the time of MRI acquisition. Mean epilepsy duration in the patient group was 5.62 ± 5.06 months. Cognitive assessment using the WISC-RC was completed for all patients, yielding mean FSIQ, VIQ, and PIQ scores within the normal range.

**Table 1:** Comparison of sociodemographic and clinical characteristics.

Variable	SeLECTS (*N* = 30)	HCs (*N* = 30)	*t/χ2*	*p*-value
	Mean ± (SD)/*n* (%)	Mean ± (SD)/*n* (%)		
Age (years)	9.26 ± 2.03	9.39 ± 1.92	−0.25	0.81^a^
Gender (M/F)	14/16 (46.7/53.3%)	18/12 (60.0/40.0%)	1.07	0.30^b^
Education (years)	2.47 ± 1.55	2.70 ± 1.75	−0.55	0.59^a^
Duration (months)	5.62 ± 5.06	/	/	/
WISC-R				
FSIQ	97.83 ± 12.84	/	/	/
VIQ	98.73 ± 14.15	/	/	/
PIQ	95.70 ± 13.59	/	/	/

Data expressed as Mean ± SD or *n* (%); FSIQ, Full Scale Intelligence Quotient; VIQ, Verbal Intelligence Quotient; PIQ, Performance Intelligence Quotient; WISC-R, Wechsler Intelligence Scale for Children-Revised; SeLECTS, self-limited epilepsy with centrotemporal spikes; HCs, healthy controls; M, male; F, female; ^a^Independent samples *t*-test; ^b^Chi-square test.

### 3.2 Voxel-based morphometry

#### 3.2.1 Gray matter volume

**Figure 1:**
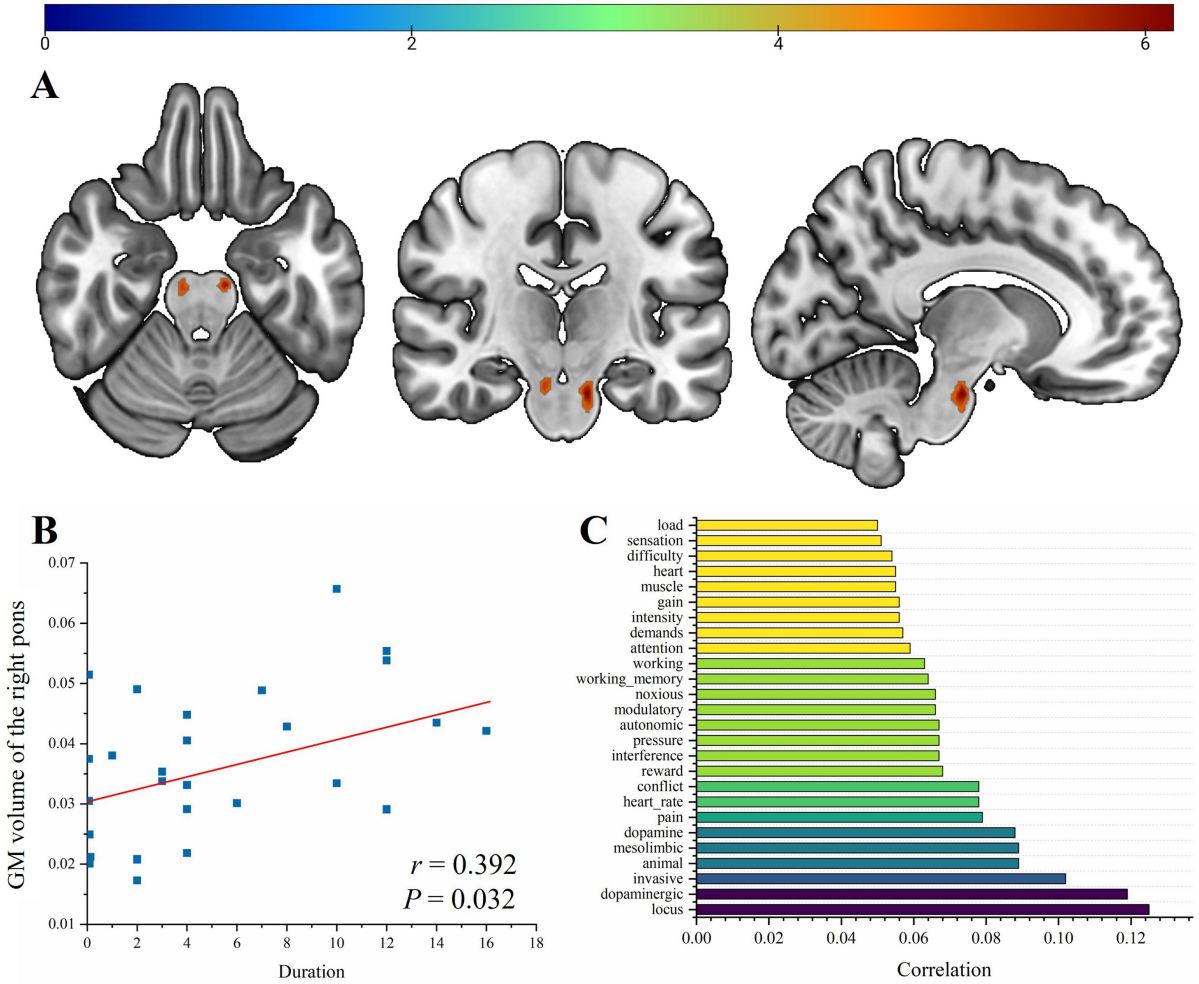
Bilateral pontine structural alterations in SeLECTS (FWE-corrected *p* < 0.05). **(A)** Bilateral pontine GM volume increases shown on brain template; **(B)** positive correlation between right pontine GM volume and disease duration (*r* = 0.392, *p* = 0.032); **(C)** horizontal bar plot displaying Neurosynth-derived terms with correlation coefficients > 0.05.

Compared with HCs, children with SeLECTS showed significantly increased GM volume in the bilateral pontine nuclei (Figure 1A). These brainstem clusters survived FWE correction at *p* < 0.05 (voxel-level). For the left pontine cluster, the peak effect was observed at approximately MNI coordinates [−10, −18, −26], with a cluster size of ~76 voxels; a similar right-sided cluster peaked near [8, −20, −22]. No regions exhibited significantly reduced GM volume in SeLECTS relative to controls at the corrected threshold. Within the SELECTS group, exploratory analysis showed a positive correlation between right pontine volume and epilepsy duration (*r* = 0.392, *p* = 0.032; Figure 1B). Exploratory meta-analytic annotation via Neurosynth identified that the spatial pattern of these clusters aligns with regions frequently associated in the literature with terms such as “locus coeruleus,” “dopaminergic” and “invasive” (Figure 1C). As specified in the methodology, these associations are interpretive and provided for generating mechanistic hypotheses (Table 2).

**Table 2:** Comparison of gray matter volume between two groups.

Region	MNI (*x, y, z*)	Cluster size (*k*)	*t/χ* ^2^	*p*-value
Left pons	(−10, −18, −26)	76	6.31	<0.001^**^
Right pons	(8, −20, −22)	50	5.51	0.012^*^

Montreal Neurological Institute (MNI) coordinates (x, y, z) refer to the peak voxel of each cluster in Montreal Neurological Institute space. Cluster Size (k) represents the number of contiguous voxels. Statistical significance was assessed using General Linear Model (GLM) analysis with Family-Wise Error (FWE) correction at the voxel level (*p* < 0.05) and a cluster extent threshold of 20 voxels.

#### 3.2.2 Structural lateralization analysis

**Figure 2:**
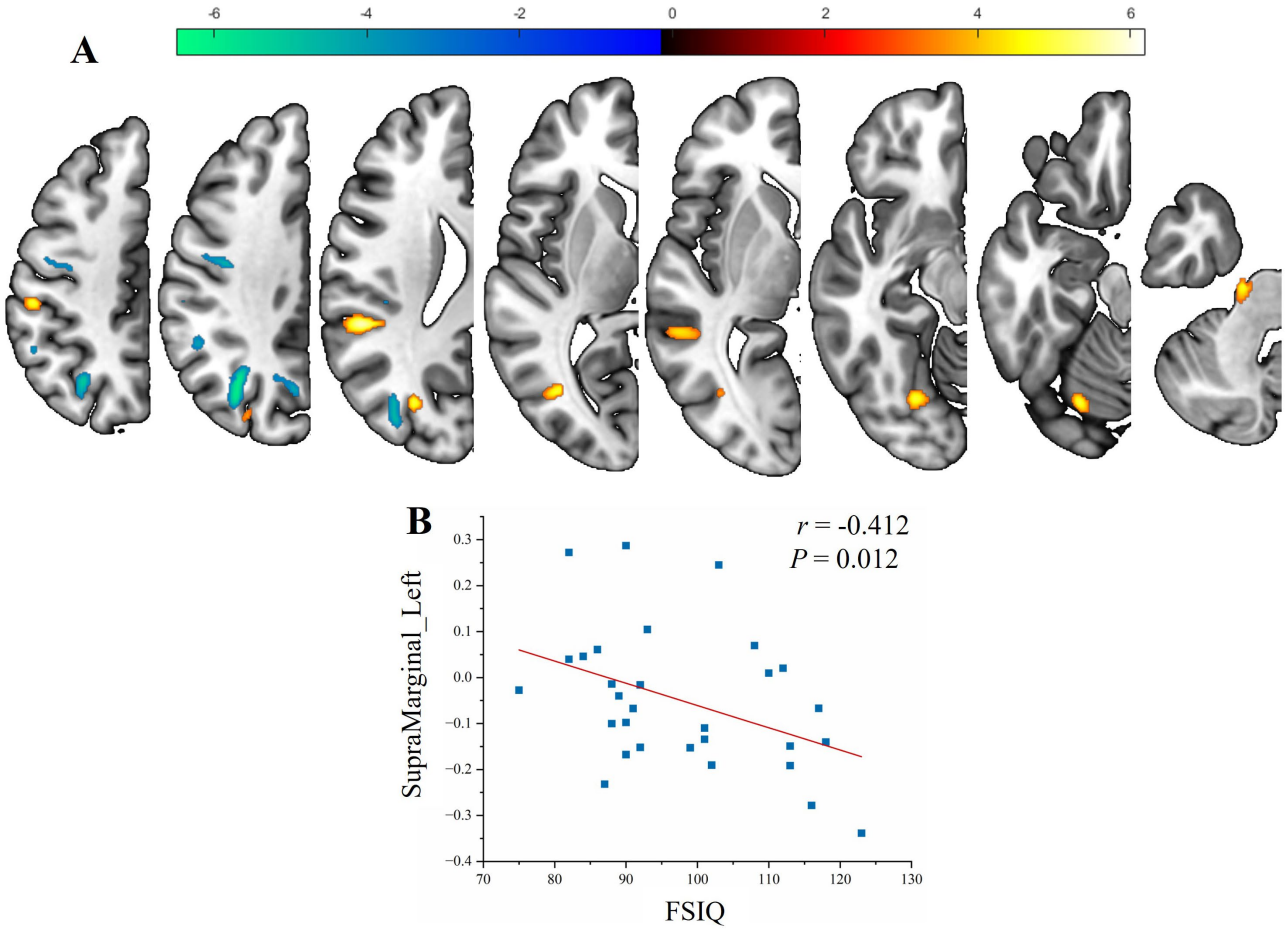
GM volume lateralization in SeLECTS and its clinical correlations. **(A)** The LI in the SeLECTS group was significantly higher than in the HCs group (warm colors) mainly in the left fusiform gyrus, supramarginal gyrus, and middle occipital gyrus; while it was significantly lower than in the HCs group (cool colors) mainly in brain regions related to the left angular gyrus. **(B)** The LI of the left supramarginal gyrus was negatively correlated with the full-scale IQ (*r* = −0.412, *p* = 0.012).

Compared with the HCs group, patients exhibited significantly altered lateralization indices (FDR-corrected *p* < 0.05; Table 3; Figure 2A). In the SeLECTS group exhibited significantly increased leftward asymmetry (greater left > right hemispheric bias) in the fusiform gyrus, supramarginal gyrus, and middle occipital gyrus. In contrast, they showed a trend toward reduced leftward asymmetry (i.e., a rightward shift) in the angular gyrus. Among these, the lateralization index of the left supramarginal gyrus was negatively correlated with the FSIQ (*r* = −0.412, *p* = 0.012; Figure 2B), suggesting that the significant increase in GM volume asymmetry in this region may be associated with lower cognitive performance.

**Table 3:** Comparison of LI between two groups.

Contrast name	Region label	Extent	*t*-value	*p*-value	MNI coordinates		
					*x*	*y*	*z*
SeLECTS>HCs	Fusiform_L	163	6.141	0.003*	−24	−72	−17
	SupraMarginal_L	171	5.720	0.004*	−50	−39	24
	Occipital Mid_L	192	5.264	0.005*	−27	−74	27
HCs > SeLECTS	Angular_L	654	6.415	<0.001**	−35	−69	32

Table shows all local maxima separated by more than 20 mm. Regions were automatically labeled using the AAL3 atlas. x, y, and z = MNI coordinates in the left–right, anterior–posterior, and inferior–superior dimensions, respectively. ^*^FDR-corrected *p* < 0.05, ^**^FDR-corrected *p* < 0.001.

### 3.3 Surface-based morphometry

#### 3.3.1 Gyrification

**Figure 3:**
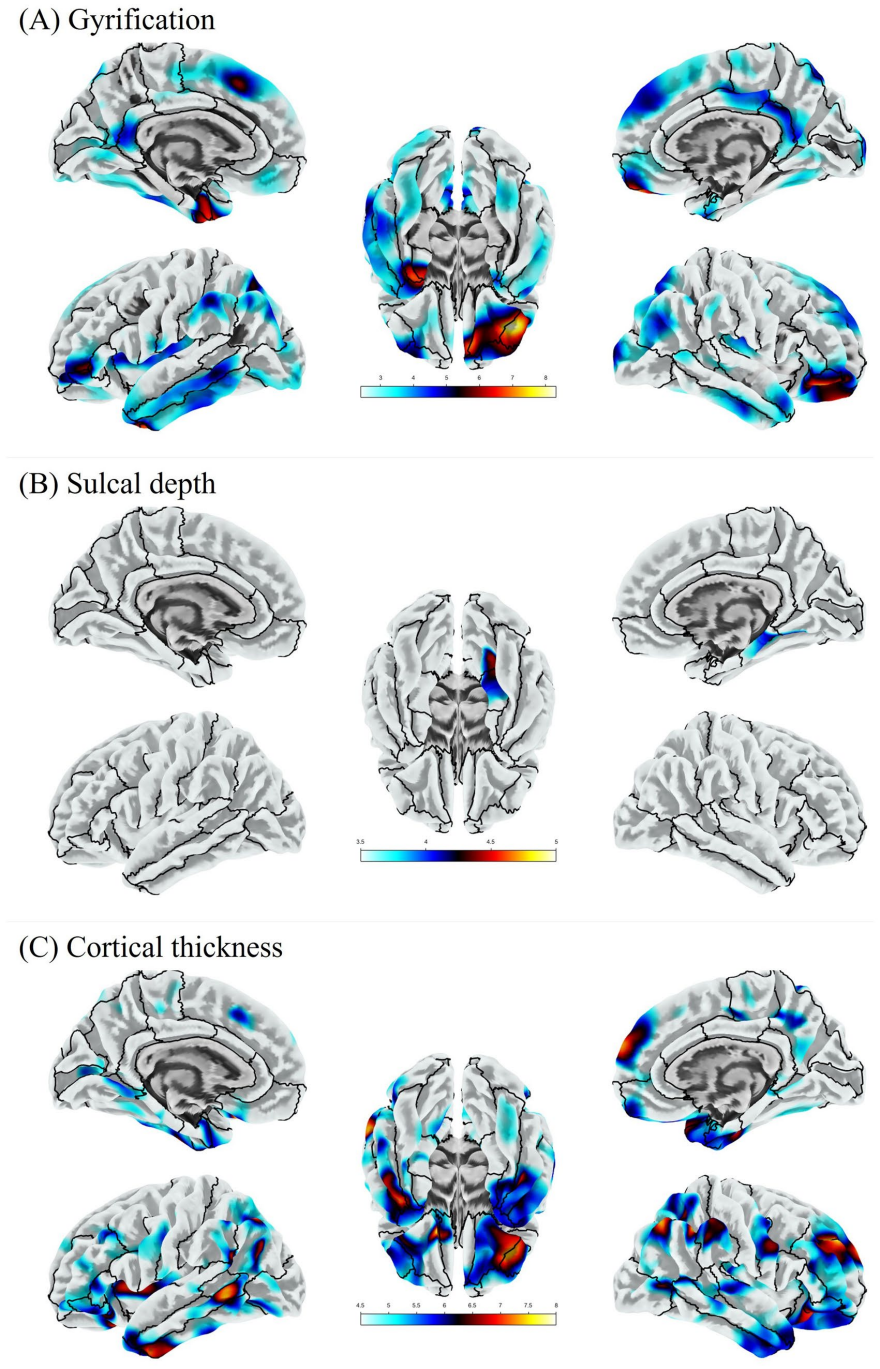
Surface-based morphometry group differences between drug-naive SeLECTS and HCs (FWE-corrected *p* < 0.05). **(A)** Increased gyrification in the right orbitofrontal region, left fusiform gyrus, and left superior frontal gyrus; **(B)** reduced sulcal depth in right lingual gyrus and parahippocampal gyrus; **(C)** cortical thinning in bilateral frontoparietal and the left temporal regions. Warm/cool colors denote increased/decreased values in patients. Cortical surfaces rendered using Desikan Killiany atlas.

SeLECTS patients exhibited regions of abnormally increased cortical folding. Specifically, gyrification index was significantly higher in the right lateral/orbitofrontal cortex, left superior frontal gyrus, and left fusiform gyrus in SeLECTS compared to controls (Figure 3A). No regions showed significantly decreased gyrification in SeLECTS at the corrected threshold. However, we note that the areas of heightened gyrification were often bordered by neighboring cortical patches with relatively lower gyrification (visible qualitatively on the cortical maps). This spatially interleaved pattern of increased and decreased gyrification suggests focal dysmaturation of cortical folding. All reported differences survived FWE correction at *p* < 0.05 (Table 4).

**Table 4:** Comparison of gyrification, depth, and thickness between two groups.

Region	HCs	SeLECTS	*t*-value	*p*-value
	Mean ± SD	Mean ± SD		
Parsorbitalis right	29.386 ± 0.846	30.849 ± 0.979	8.325	<0.001^**^
Lateralorbitofrontal right	26.949 ± 1.129	27.792 ± 0.956	7.072	<0.001^**^
Medialorbitofrontal right	28.764 ± 0.712	29.483 ± 0.944	5.445	<0.001^**^
Fusiform left	28.154 ± 0.873	29.299 ± 1.351	6.868	<0.001^**^
Entorhinal left	28.697 ± 0.934	29.466 ± 1.016	6.155	<0.001^**^
Superiorfrontal left	27.903 ± 0.583	28.622 ± 0.854	6.001	<0.001^**^
Lingual right	13.255 ± 1.464	12.211 ± 1.521	4.755	0.023^*^
Parahippocampal right	14.807 ± 2.158	12.416 ± 1.916	4.662	0.030^*^
Rostralmiddlefrontal right	2.802 ± 0.095	2.569 ± 0.143	7.969	<0.001^**^
Lateralorbitofrontal left	1.835 ± 0.286	1.463 ± 0.185	7.784	<0.001^**^
Parsorbitalis right	2.778 ± 0.113	2.587 ± 0.113	7.618	<0.001^**^
Superiorfrontal right	2.401 ± 0.106	2.230 ± 0.133	7.472	<0.001^**^
Middletemporal left	3.015 ± 0.123	2.722 ± 0.173	7.418	<0.001^**^
Inferiorparietal right	2.856 ± 0.134	2.565 ± 0.203	6.929	<0.001^**^
Inferiorparietal left	2.819 ± 0.101	2.604 ± 0.155	6.847	<0.001^**^

Data expressed as Mean ± SD; FWE, family-wise error correction; SeLECTS, self-limited epilepsy with centrotemporal spikes; HC, healthy control.^*^FWE-corrected *p* < 0.05, ^**^FWE-corrected *p* < 0.001.

**Figure 4:**
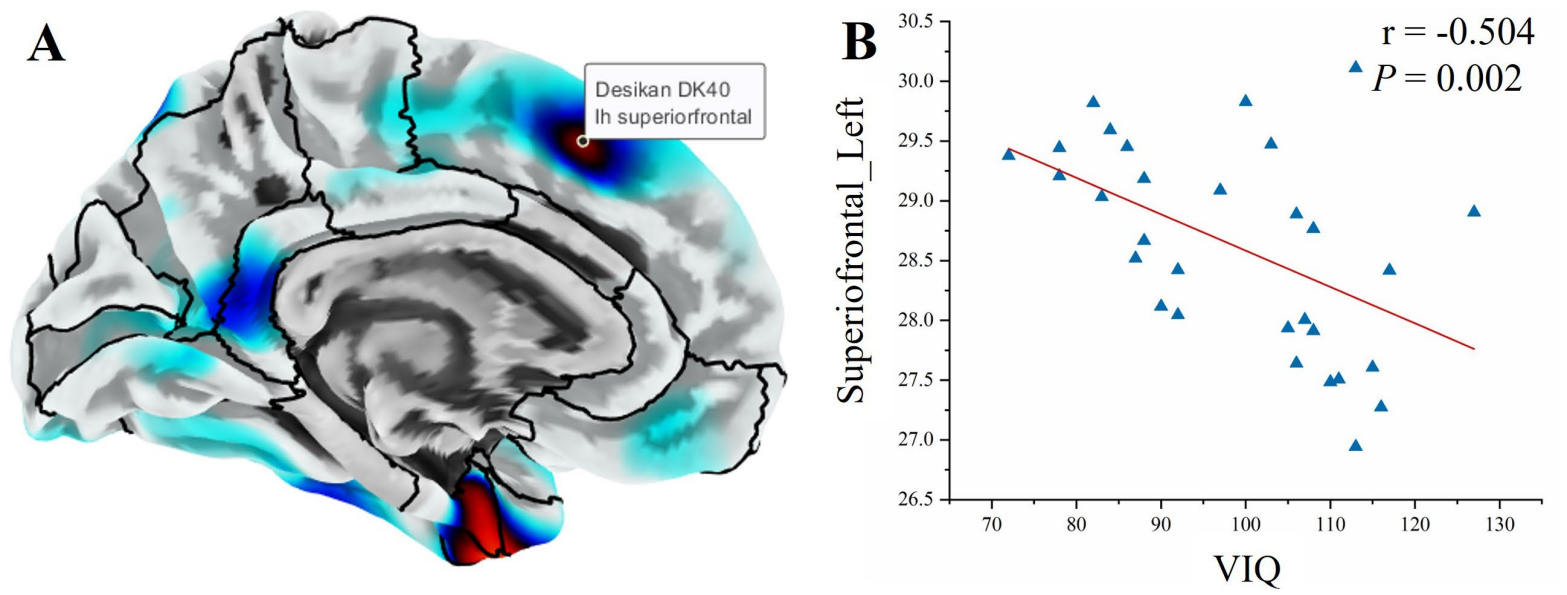
Correlations between gyrification and cognitive variables in SeLECTS. **(A)** Anatomical localization of the left superior frontal gyrus. **(B)** Scatterplot showing a negative correlation between left superior frontal gyrification and VIQ (*r* = -0.504, *p* = 0.002).

In the SeLECTS group, gyrification of the left superior frontal gyrus was negatively correlated with VIQ (*r* = −0.504, *p* = 0.002) (Figure 4).

#### 3.3.2 Sulcal depth

Sulcal depth was significantly reduced in the SeLECTS group in the right lingual gyrus and right parahippocampal gyrus (Figure 3B), indicating shallower-than-normal sulci in these medial temporal regions. No other sulcal depth differences were observed. All differences survived FWE correction at *p* < 0.05 (Table 4).

#### 3.3.3 Cortical thickness

The SeLECTS group had significantly reduced cortical thickness in widespread regions of the frontal, temporal, and parietal lobes (Figure 3C). In the left hemisphere, the lateral orbitofrontal gyrus, inferior parietal lobule (including supramarginal gyrus), and middle temporal gyrus were thinner in patients than in controls. In the right hemisphere, thinner cortex was observed in the pars orbitalis of the inferior frontal gyrus, rostral middle frontal gyrus, superior frontal gyrus, and inferior parietal lobule. All cortical thickness differences remained significant after FWE correction (*p* < 0.05; Table 4). No cortical region was significantly thicker in SeLECTS than in controls. We did observe small clusters with nominally increased cortical thickness in the patient group (e.g., in portions of the right insula and anterior cingulate), but these did not survive FWE correction. Thus, cortical thinning was the predominant pattern, consistent with an overall accelerated or aberrant pruning process in SeLECTS.

**Figure 5:**
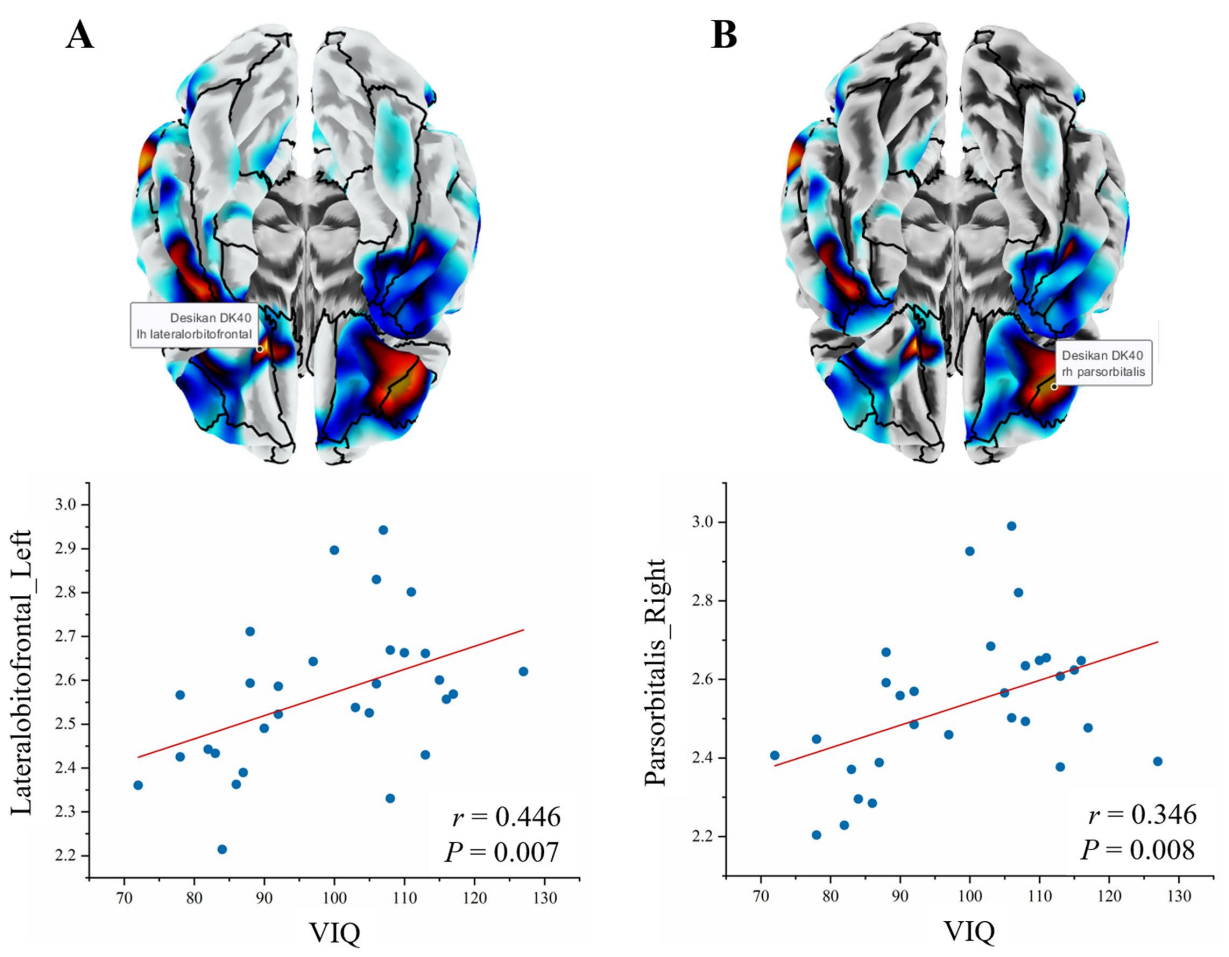
Correlations between cortical thickness and clinical variables. **(A)** Left lateral orbitofrontal gyrus thickness positively correlated with VIQ (*r* = 0.446, *p* = 0.007); **(B)** Right pars orbitalis thickness showed positive association with VIQ (*r* = 0.346, *p* = 0.008). Red regression lines with 95% confidence intervals represent the linear fit. Cortical surfaces rendered using Desikan-Killiany atlas.

Within the SeLECTS group, cortical thickness of the left lateral orbitofrontal gyrus (*r* = 0.446, *p* = 0.007) and right pars orbitalis (*r* = 0.346, *p* = 0.008) was positively correlated with VIQ (Figure 5).

## 4 Discussion

This study investigated the structural brain characteristics of drug-naïve children with SeLECTS using a multimodal imaging approach combining SBM and VBM. The results revealed widespread cortical and subcortical structural alterations in patients compared to healthy controls, particularly increased cortical sulcation in the frontotemporal regions and generalized cortical thinning. Additionally, we observed increased GM volume in the bilateral pontine nuclei. The cortical findings provide strong support for the “developmental dysregulation” hypothesis, while the newly discovered subcortical gray matter structural variations suggest a holistic adaptive mechanism of the nervous system in response to chronic epileptiform activity.

We observed significantly increased gyrification in the orbitofrontal cortex and left superior frontal gyrus. In normal pediatric development, cortical gyrification undergoes a simplification process characterized by synaptic pruning and the mechanical tension of white matter expansion—a process often referred to as tension-based morphogenesis (29–31). The persistence of “hyper-gyrification” in drug-naïve patients suggests a disruption or delay in this maturation process. Interestingly, we found that higher gyrification in the superior frontal gyrus was associated with lower Verbal IQ scores. This negative correlation implies that the “extra” cortical folding is pathological rather than advantageous, likely reflecting a failure of the synaptic pruning mechanisms required to optimize communication between cortical columns (32, 33). This pattern of “delayed simplification” in the frontal lobes may underlie the deficits in executive function and complex language processing frequently observed in these children.

A key finding of the SBM analysis was widespread cortical thinning, particularly in bilateral frontal, temporal, and parietal regions. While cortical thinning is often associated with atrophy in degenerative diseases, in the context of childhood epilepsy, this may reflect abnormal pruning or alterations in dendritic structure driven by epileptiform activity (34, 35). Interestingly, when using VBM analysis, we did not observe corresponding reductions in gray matter volume in these regions. This discrepancy may arise because cortical thinning is offset by concurrent increases in cortical surface area or intensified gyrification, thereby preserving total volume (36). Furthermore, the inherent spatial smoothing in VBM may render it less sensitive to subtle thickness changes (23). This discrepancy emphasizes the necessity of utilizing surface-based techniques like SBM to detect subtle architectural shifts that VBM may miss. The positive correlation between cortical thickness in the left lateral orbitofrontal gyrus and the right orbital cortex with verbal IQ highlights the functional vulnerability of these regions. These hub areas are central to language and executive networks. Thinning in these regions in children with SeLECTS may reflect a reduction in the complexity of the neuropil, potentially stemming from the disruptive effects of interictal spikes on local synaptic stability, consistent with the common neuropsychological profile of SeLECTS (37–39). This thinning pattern differs from the more widespread or motor-cortex-focused thinning commonly seen in generalized epilepsy syndromes (35, 40). In SeLECTS, the focal nature of the discharges appears to preferentially affect “adjacent” association cortices, interfering with the maturation of auditory-language and executive hubs. This prominent temporal lobe involvement likely reflects the focal nature of centrotemporal spikes and their impact on the auditory-language network (41, 42). Therefore, thinning in these regions may underlie the common language and memory deficits observed in patients with SeLECTS (5).

Furthermore, the observed reduction in cortical depth in the right lingual gyrus and right parahippocampal gyrus further emphasizes the impeded normal expansion of the medial temporal lobe in SeLECTS. Sulcal depth is closely related to the growth of underlying white matter and the tension of long-range association fibers (43). The lingual and parahippocampal gyri play key roles in memory consolidation and spatial navigation (44), and their involvement in epilepsy-related cognitive impairment is well-documented (34). The reduced cortical depth we found in this region aligns with previous studies showing altered folding complexity in the medial temporal lobe of patients with drug-resistant JME. These structural abnormalities may reflect disruptions in synaptic pruning or dendritic branching during critical periods of brain development and could be exacerbated by recurrent epileptiform activity (45). The presence of shallower sulci than normal in these regions crucial for memory and visuospatial processing indicates that the neurodevelopmental abnormalities in SeLECTS extend far beyond the Rolandic (centrotemporal) cortex, involving deep structures responsible for complex cognitive functions. The alterations in gyrification and depth may stem from a combination of factors, including genetic susceptibility, neurodevelopmental delays, and the pathological effects of centrotemporal spikes (31). Epilepsy-related disruption of cortical–subcortical circuits may lead to abnormal structural covariance networks, as our previous research has demonstrated excessive folding in the perisylvian cortex and a shift of network hub nodes toward sensorimotor and temporal regions in SeLECTS patients (46). These structural abnormalities may, in turn, affect functional connectivity, leading to the cognitive impairments observed in this study.

Lateralization analysis revealed a transition to significantly increased asymmetry in the perisylvian regions. These areas are known semantic processing hubs and key components of the default mode network (47–49). In typical right-handed development, the patients’ strong gray matter volume asymmetry in these regions may indicate impaired normal functional specialization of the brain. The correlation between increased supramarginal gyrus asymmetry and poorer cognitive performance provides a structural basis for the cognitive vulnerability in these children (50). The supramarginal gyrus is a critical hub for semantic processing and visual word recognition; enhanced structural “lateralization” in this region may be an intrinsic mechanism of specific language impairment and is also related to the attentional difficulties frequently reported in this syndrome (51). These findings are consistent with functional MRI studies showing atypical language lateralization and reduced network efficiency in children with focal epilepsy (52).

One of the most distinctive findings of our VBM analysis was the bilateral volume increase in a pontine cluster. This finding is particularly significant because the pontine nuclei serve as the primary relay station of the corticopontocerebellar pathway, responsible for transmitting a vast amount of motor and cognitive instructions from the cerebral cortex to the cerebellum (53, 54). The positive correlation between the volume of the right pons and epilepsy duration suggests that this subcortical alteration is not an innate feature but rather a possible consequence of chronic seizure activity. The cerebellum is no longer viewed as a purely motor organ, it is now recognized as a critical “gatekeeper” for emotional and cognitive processing (55, 56). The increased volume of the pontine nuclei may reflect “use-dependent” plasticity within the relay nuclei. When the Rolandic cortex generates excessive interictal discharges, the pontine nuclei may experience heightened synaptic activity, leading to structural remodeling. Although this reorganization might be protective against seizure spread, it could also disrupt the fine coordination of motor and cognitive information, providing a structural basis for the subtle “clumsiness” and executive dysfunction observed in these children. Therefore, the involvement of the pontine nuclei in SeLECTS reflects a system-level response where subcortical structures attempt to buffer pathological cortical activity. Although our VBM cluster is centered in the pontine region, standard 3 T T1-weighted MRI lacks the spatial resolution and specific tissue contrast required to precisely localize these findings to individual sub-nuclei, such as the locus coeruleus (LC). The exact mechanistic driver of this pontine hypertrophy is unclear. As a purely exploratory hypothesis, chronic epileptiform activity might induce broader adaptive structural changes within brainstem neuromodulatory networks (57). For instance, noradrenergic pathways originating in the brainstem play a known role in suppressing epileptic activity, and theoretically, sustained engagement of these systems could lead to regional structural remodeling (58, 59). We emphasize, however, that this interpretation remains highly speculative, our current data do not support precise anatomical localization or definitive mechanistic conclusions regarding specific pontine nuclei.

Several limitations must be addressed. First, the cross-sectional nature of the study prevents us from determining the causal direction of the observed changes. Whether the cortical thinning precedes the onset of CTS or is a consequence of repeated network disruption remains unclear. Second, our sample size of 30 patients, while standard for drug-naïve pediatric epilepsy research, is relatively small. Larger, multi-center cohorts are needed to confirm these findings. Third, the use of the WISC-RC, while justified in our regional context, may complicate comparisons with international studies using the WISC-V. Future research should aim to use the most recent standardized instruments available. Finally, our interpretation of the brainstem findings is limited by the lack of neuromelanin-sensitive or high-resolution brainstem-specific sequences. Future studies should employ molecular or specific tissue-contrast imaging to definitively evaluate the LC-NE system.

Drug-naïve children with SeLECTS exhibit a widespread pattern of structural brain deviations that extend well beyond the Rolandic cortex. Our results reveal a syndrome defined by a complex interplay between cortical dysmaturation—characterized by hyper-gyrification and thinning—and subcortical adaptation in the brainstem. The identified structural markers, particularly the atypical asymmetry of perisylvian hubs and the hypertrophy of pontine clusters, offer potential biomarkers for identifying children at risk for persistent cognitive impairment. This research provides a new framework for understanding the neurodevelopmental trajectory of SELECTS, emphasizing that the “self-limited” nature of the seizures does not imply a “benign” impact on the developing brain.

## Data Availability

The original contributions presented in the study are included in the article/supplementary material, further inquiries can be directed to the corresponding author.

## Appendix: Supplementary methods

To explore the potential cognitive relevance of regions showing significant VBM differences, meta-analytic decoding was performed using the Neurosynth platform (https://neurosynth.org/decode/). Group-level statistical maps were uploaded to the Neurosynth Decoder to identify cognitive terms associated with the observed spatial patterns. Only non-anatomical cognitive terms with correlation coefficients greater than 0.05 were retained. This analysis was exploratory and intended to aid interpretation rather than to establish definitive functional attribution. Specifically, the Neurosynth database combines activation data from thousands of published MRI studies to create term-based meta-analytic activation maps. By correlating our group-level VBM map with these meta-analytic term maps, we identify the cognitive or anatomical labels most strongly associated with our observed structural differences.
